# Association between Long-Term Changes in Dietary Percentage of Energy from Fat and Obesity: Evidence from over 20 Years of Longitudinal Data

**DOI:** 10.3390/nu14163373

**Published:** 2022-08-17

**Authors:** Chenlu Wu, Baibing Mi, Wanrong Luo, Binghua Chen, Jiao Ma, Hao Huang, Qian Zhang, Yaqiong Wang, Heng Liu, Binguo Yan, Fangyao Chen, Leilei Pei, Ruru Liu, Xueying Qin, Duolao Wang, Hong Yan, Yaling Zhao

**Affiliations:** 1 Department of Epidemiology and Biostatistics, School of Public Health, Xi’an Jiaotong University Health Science Center, No. 76, Yanta West Road, Xi’an 710061, China; 2 Xi’an Center for Disease Control and Prevention, No. 599, Xiying Road, Xi’an 710054, China; 3 Department of Epidemiology and Biostatistics, School of Public Health, Peking University, No. 38, Xueyuan Road, Haidian District, Beijing 100191, China; 4 Department of Clinical Sciences, Liverpool School of Tropical Medicine, Pembroke Place, Liverpool L3 5QA, UK

**Keywords:** percentage of energy from fat, obesity, longitudinal data, trajectory

## Abstract

Objectives: This study assessed the associations between long-term trajectories of percentage of energy from fat (PEF) and obesity among Chinese adults. Methods: Longitudinal data collected by the China Health and Nutrition Survey from 1991 to 2015 were analyzed. A body mass index ≥28.0 was defined as general obesity. Participants’ baseline PEF levels were categorized as lower than the recommendation of the Chinese Dietary Guideline (<20%), meeting the recommendation (20–30%), and higher than the recommendation (>30%). Patterns of PEF trajectories were identified by latent class trajectory analysis for overall participants and participants in different baseline PEF groups, respectively. Cox proportional hazards regression models with shared frailty were used to estimate associations between PEF and obesity. Results: Data on 13,025 participants with 72,191 visits were analyzed. Four patterns of PEF trajectory were identified for overall participants and participants in three different baseline PEF groups, respectively. Among overall participants, compared with “Baseline Low then Increase Pattern” (from 12% to 20%), participants with “Baseline Normal-Low then Increase-to-High Pattern” (from 20% to 32%) had a higher hazard of obesity (hazard ratio (HR) and 95% confident interval (CI) at 1.18 (1.01–1.37)). Compared with the “Stable Pattern” group (stable at around 18% and 22%, respectively), participants with “Sudden-Increase Pattern” (from 18% to 30%) in the baseline group whose PEF levels were lower than the recommendation and those with “Sudden-Increase then Decrease Pattern” (rapidly increased from 25% to 40%, and then decreased) in the baseline group who met the recommendation had higher hazards of obesity (HRs and 95% CIs being 1.65 (1.13–2.41) and 1.59 (1.03–2.46), respectively). Conclusions: Adults with a trajectory that involved a sudden increase to a high-level PEF had a higher risk of general obesity. People should avoid increasing PEF suddenly.

## 1. Introduction

Obesity, which is defined as “a chronic complex disease defined by excessive adiposity that impairs health” in the International Classification of Diseases 11 (ICD-11) [1], has become a worldwide challenge over the past decades. Since 1980, the prevalence of obesity has doubled in more than 70 countries and has continuously increased in most countries [2]. In China, obesity has also become a significant public health problem. The prevalence of obesity among Chinese adults increased from 4.0% in 1993 to 15.7% in 2015 [3,4]. Obesity is an established risk factor for several kinds of chronic non-communicable diseases, such as cardiovascular diseases [5,6], type 2 diabetes [5,6], chronic kidney disease [6], and cancers [7]. Those obesity-related diseases have led to severe health threats and disease burden. In 2015, high body mass index (BMI), a surrogate marker of adiposity [1], contributed to 4 million deaths worldwide [2].

The decrease in the plant-characterized traditional Chinese diet, the increase in the Western diet, the decline in physical activity, and the rise in sedentary activity contributed to the rapid increase in overweight and obesity in China [8]. With the dietary pattern transforming from a traditional Chinese diet to a Western diet, the daily dietary fat intake and the percentage of energy from fat (PEF) have increased [9]. However, the relationship between fat intake and obesity is under-determined. A study reported that the high-fat diet was a risk factor for the prevalence of obesity and chronic diseases in China [10]. Another study among young and middle-aged US adults showed that a high-fat diet was associated with a high BMI [11]. On the contrary, other studies observed that total energy intake, rather than a high PEF, played an essential role in obesity [12,13].

Most of the previous observational studies about the relationship between fat intake and obesity were cross-sectional studies [11,14,15,16], or cohort studies [17,18,19,20,21,22,23] that only used the baseline dietary intake or dietary intake change between the baseline survey and a follow-up assessment. In the studies [11,14,15,16,17,22,23] which only had a dietary assessment at one time, the cumulative and longitudinal effect of dietary fat intake and the effect of change in fat intake could not be estimated. In the cohort studies [18,19,20,21] using dietary assessments at baseline and a follow-up time, the range of the change in fat intake between the baseline and the follow-up time was estimated, but the effect of the fat intake trajectory was not estimated. Therefore, using data from a large nationwide longitudinal study, the China Health and Nutrition Survey (CHNS), we aimed to assess the effect of the long-term trajectory of PEF on obesity among Chinese adults by performing a latent class trajectory analysis (LCTA).

## 2. Methods

### 2.1. Study Design and Participants

In the present study, we used data collected by the CHNS from 1991 to 2015. The CHNS is an ongoing longitudinal study that aims to collect representative data on critical public health risk factors, health outcomes, and nutritional status of the Chinese population [24]. CHNS was initiated in 1989 and has been followed-up every two to four years. By now, there have been ten waves of surveys, which were conducted in 1989, 1991, 1993, 1997, 2000, 2004, 2006, 2009, 2011, and 2015, respectively. Participants in the CHNS were selected from nine provinces in China. Details about the design and procedures of the CHNS have been reported by Popkin et al. [24].

**Figure 1:**
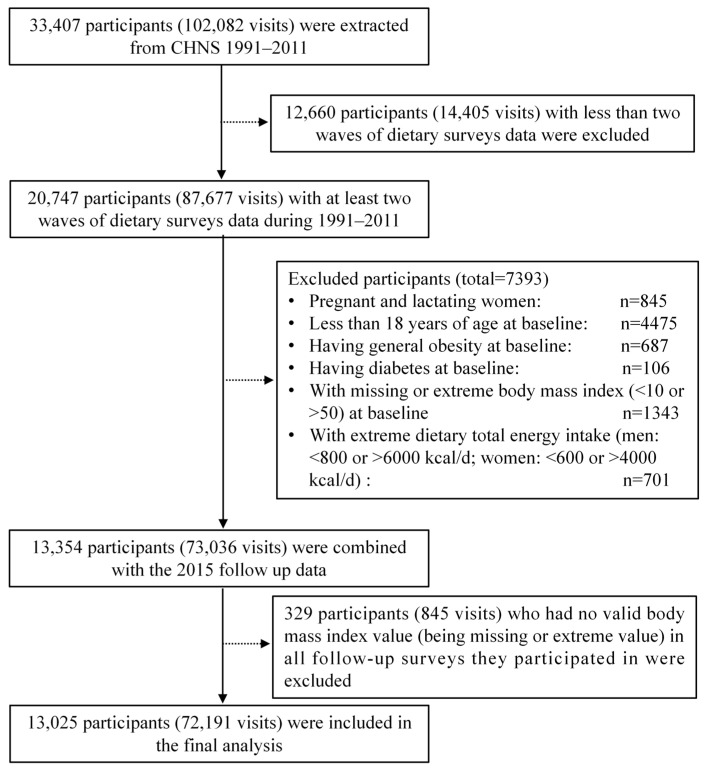
Flow diagram of participants in the study.

Our analysis used data from nine waves of the CHNS (1991–2015), as the 1989 wave did not collect dietary assessment data of all participants. Dietary data of the 2015 wave has not yet been wholly opened; only the opened data, such as health outcome information in 2015, were used. Pregnant and lactating women and participants who were younger than 18 years old at baseline had less than two waves of dietary data, had extreme dietary total energy intake (<800 kcal/d or >6000 kcal/d for men; <600 kcal/d or >4000 kcal/d for women) [25], had obesity or diabetes at baseline, had a missing or extreme BMI (<10 or >50) at baseline, and had no valid BMI (being missing or extreme value) in all follow-up surveys they participated in were excluded from the analysis. Finally, a total of 72,191 visits contributed by 13,025 participants were included in the final analysis. The flow diagram of the selection of the study participants is shown in Figure 1.

### 2.2. Definition of Follow-Up in the Study

Participants included in this analysis were followed prospectively from the time of their first visit to the CHNS. As participants in this open cohort might join or leave the cohort at any wave, participants who were lost to follow-up in one wave could rejoin the survey in the next waves. We defined the baselines of the study population as their first visits to the CHNS between 1991 and 2011. The duration of follow-up was defined as the period from the first visit to the latest visit that the participant attended or the occurrence of general obesity (BMI ≥ 28.0), death, or other loss to follow-up from the CHNS.

### 2.3. Assessment of Dietary Intake

In each wave of the CHNS, three consecutive 24 h dietary recalls, namely two weekdays and one weekend day, were used to collect dietary intake data at the individual level, and household food consumption data during the same three-day period were also collected. Each individual’s daily macronutrient (g/d) intake and total energy intake (kcal/d) were calculated by using the Chinese Food Composition Table. The percentage of energy from fat was calculated by using the energy from fat divided by the daily total energy intake. The amount of energy from fat was calculated as follows: fat intake (g) × 9 kcal (37 kJ). According to the recommended PEF (20–30% total energy from fat) by the Chinese Dietary Guidelines [26], participants’ baseline PEF levels were categorized as lower than the recommendation (<20%), meeting the recommendation (20–30%), and higher than the recommendation (>30%).

### 2.4. Assessment of Outcomes

Measurements of weight and height of participants were collected by trained health workers by using standard protocols at each wave [24,27,28]. Weight was measured without shoes and wearing lightweight clothing to the nearest 0.1 kg, using a calibrated scale (SECA 880) [27,28]. Height was measured to the nearest 0.1 cm without shoes, using a portable SECA stadiometer (SECA 206 wall-mounted metal tapes) [27,28]. BMI was calculated as weight (kg) divided by the square of height (m^2^). BMI was divided into four categories based on the cutoffs suggested by the Working Group on Obesity in China (underweight: BMI < 18.5; normal: 18.5 ≤ BMI < 24.0; overweight: 24.0 ≤ BMI < 28.0; and obesity: BMI ≥ 28.0) [29]. The primary outcome was the time from baseline to the first occurrence of obesity during the follow-up.

### 2.5. Assessment of Covariates

Participants’ sociodemographic characteristics (including age, gender, nationality, education level, marital status, family economic level, region, and community type) and lifestyle factors (smoking, drinking, and physical activity habits) were collected with the questionnaire in each wave. Nationality was categorized as Han and other nationalities. Marital status was categorized into three groups: married, unmarried, and divorced/separate/widowed. Education level was divided into four categories: illiteracy, primary school, middle school, and high school and above. Participants’ per capita annual family income at baseline was divided into three levels (high, middle, and low) according to the per capita annual family income tertiles. Community type included four categories: city, suburb, town, and village. Region was categorized into four groups: Northeast (Heilongjiang and Liaoning provinces), East Coast (Shandong and Jiangsu provinces), Central (Henan, Hubei, and Hunan provinces), and Western (Guangxi autonomous region and Guizhou province). Participants were divided into current smokers and non-smokers, and current drinkers and non-drinkers, according to their current smoking and drinking status, respectively. Participants’ physical activity level was classified into three categories (light, medium, and heavy) based on their self-reported activities, including occupational, domestic, transportation, and leisure-time physical activities.

### 2.6. Statistical Analysis

Continuous variables were presented as mean ± standard deviation (SD). Categorical variables were presented as frequencies (%). Analysis of variance, Chi-square tests, and rank sum tests were used to compare continuous, categorical, and ordinal variables, respectively. Cox proportional hazards regression models with shared frailty were used to estimate the hazard ratio (HR) and its 95% confidence interval (CI) of the occurrence of obesity in different PEF groups. Model 1 was the crude model with the categorized baseline PEF level or trajectory of PEF change as the only risk factor and family as the random effect. In Model 2, we further adjusted for sociodemographic factors (gender, age, marital status, nationality, education, community type, region, and family economic level). In Model 3, we further adjusted for lifestyle factors, including smoking, drinking, and physical activity. In Model 4, we further adjusted for dietary energy intake.

The trajectories of PEF were estimated with LCTA, a method used to identify unobserved trajectory classes in epidemiological data [30,31], for overall participants and participants in three groups with different baseline PEF, respectively. The best-fit models were chosen based on Bayesian Information Criteria and practical significance of the change in PEF. According to the estimated trajectories of PEF, participants were classified into the trajectory groups with the highest posterior probability [31]. Furthermore, to evaluate the effect of missing covariates data on the association between PEF and obesity, sensitivity analyses were performed on the samples with missing values imputed by using the multiple imputation method.

All statistical tests were two-sided, and statistical significance was set at *p* < 0.05. Statistical analyses were performed by using SAS 9.4 (SAS Institute, Chicago, IL, USA). The TRAJ procedure in SAS 9.4 was used to conduct LCTA, and PROC MI was used to perform the multiple imputation of missing values.

## 3. Results

### 3.1. General Characteristics of Participants in the Study

A total of 13,025 participants with 72,191 visits were analyzed in this study. Their average visits were 5.54 ± 2.22 times, and the average follow-up period was 13.96 ± 7.17 years. Among all the analyzed participants, 4894 (37.6%) participants’ baseline PEF levels were lower than the recommended PEF for adults by the Chinese Dietary Guideline (<20%), 4105 (31.5%) were higher than the recommendation (>30%), and only 4026 (30.9%) met the recommendation (20–30%). Participants’ general characteristics by the PEF level at baseline are presented in Table 1.

### 3.2. PEF Trajectory Patterns of Overall Participants

During the follow-up period, participants’ average PEF increased from 24.9% in 1991 to 33.6% in 2011. The proportion of participants whose PEF levels were higher than the recommendation (>30%) increased from 21.9% in 1991 to 60.1% in 2011.

**Figure 2:**
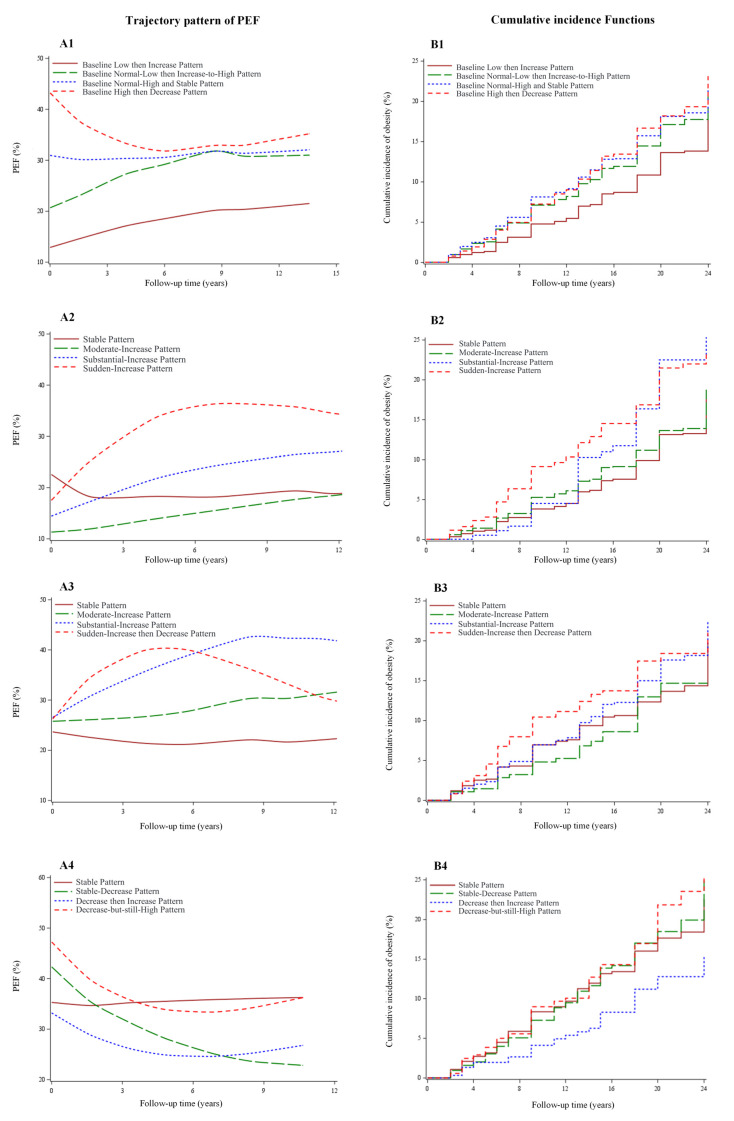
Change trajectories of the percentage of energy from fat and the cumulative incidence of general obesity among overall participants and participants in different baseline percentage of energy from fat groups. PEF: percentage of energy from fat. (**A1**) Change trajectories of PEF among overall participants. (**A2**) Change trajectories of PEF among participants with PEF level lower than the recommendation (<20%) at baseline. (**A3**) Change trajectories of PEF among participants with recommended level of PEF (20–30%) at baseline. (**A4**) Change trajectories of PEF among participants with PEF level higher than the recommendation (>30%) at baseline. (**B1**) Cumulative incidence of general obesity of different patterns of change trajectory of PEF among overall participants. (**B2**) Cumulative incidence of general obesity of different patterns of change trajectory of PEF among participants with PEF level lower than the recommendation (<20%) at baseline. (**B3**) Cumulative incidence of general obesity of different patterns of change trajectory of PEF among participants with recommended level of PEF (20–30%) at baseline. (**B4**) Cumulative incidence of general obesity of different patterns of change trajectory of PEF among participants with PEF level higher than the recommendation (>30%) at baseline.

Among all the analyzed participants, most of them showed a rising tendency in PEF during the follow-up period. According to the baseline level and change speed of PEF, four patterns of PEF change trajectory were identified (Figure 2(A1)). The first pattern of PEF change trajectory, characterized by an increase from 12% to 20% in PEF during the follow-up period was named the “Baseline Low then Increase Pattern” (*n* = 3649, 28.0%). The second pattern, characterized by an increase from 20% to 32% in PEF during the follow-up period, was named the “Baseline Normal-Low then Increase-to-High Pattern” (*n* = 4376, 33.6%). The third pattern, which was characterized by staying stable at around 30% in PEF during the follow-up period, was named “Baseline Normal-High and Stable Pattern” (*n* = 3733, 28.7%). The fourth pattern, having a decrease from 42% to 33% in PEF, was named “Baseline High then Decrease Pattern” (*n* = 1267, 9.7%).

### 3.3. PEF Trajectory Patterns of Participants with Different Baseline PEF Levels

The group of participants whose PEF levels at baseline were lower than the recommendation (<20%) showed a rising tendency in PEF during the follow-up period. According to the change speed and amplitude, four patterns of PEF change trajectory were identified (Figure 2(A2)). The first pattern of trajectory, characterized by staying stable at around 18% in PEF during the follow-up period was named the “Stable Pattern” (*n* = 1464, 29.9%). The second pattern, characterized by an increase from 11% to 20% in PEF during the follow-up period, was named the “Moderate-Increase Pattern” (*n* = 2350, 48.0%). The third pattern, characterized by an increase from 15% to 25% in PEF during the follow-up period, was named the “Substantial-Increase Pattern” (*n* = 891, 18.2%). The fourth pattern, having an increase from 18% to 30% in PEF, was named the “Sudden-Increase Pattern” (*n* = 189, 3.9%).

The group of participants who met the PEF recommendation (20–30%) at baseline also showed a rising tendency in PEF during the follow-up period. Among this group, four patterns of change trajectory were also identified: “Stable Pattern” (*n* = 279, 6.9%; PEF stayed stable at around 22% during the follow-up period), “Moderate-Increase Pattern” (*n* = 2442, 60.7%; PEF increased from 25% to 32%), “Substantial-Increase Pattern” (*n* = 474, 11.8%; PEF increased from 25% to 40%), and “Sudden-Increase then Decrease Pattern” (*n* = 830, 20.6%; PEF rapidly increased from 25% to 40%, and then decreased) (Figure 2(A3)).

The group of participants whose PEF levels at baseline were higher than the recommendation (>30%) showed a decreasing tendency in their PEF during the follow-up period. Four patterns of change trajectory were also identified: “Stable Pattern” (*n* = 507, 12.4%; PEF kept stable at around 35% during the follow-up period), “Stable-Decrease Pattern” (*n* = 2264, 55.2%; PEF decreased from 42% to 22%), “Decrease then Increase Pattern” (*n* = 303, 7.4%; PEF decreased from 33% to 25% and then rose), and “Decrease-but-still-High Pattern” (*n* = 1031, 25.1%; PEF declined from 48% to 33%) (Figure 2(A4)).

### 3.4. Association between Pattern of PEF Trajectory and the Risk of Obesity

During an average follow-up of 13.96 years, 1571 (12.1%) participants became obese. The cumulative incidence of general obesity was 12.8%, 12.0%, and 11.3% among participants who were lower than, met, or were higher than the recommended level of PEF at baseline, respectively. Compared with the group who met the recommendation at baseline, neither the hazard of obesity of the lower PEF group (HR and 95% CI: 1.00 (0.88–1.14)) nor that of the higher PEF group (HR and 95% CI: 0.96 (0.84–1.10)) was statistically significant (Table 2).

Hazards of obesity of adults with different patterns of PEF trajectory among overall participants are presented in Table 3 and Figure 2(B1). Compared with the “Baseline Low then Increase Pattern”, the “Baseline Normal-Low then Increase-to-High Pattern” showed a positive association with obesity (HR and 95% CI: 1.18 (1.01–1.37)). However, no statistically significant association with obesity was observed in the “Baseline Normal-High and Stable Pattern” (HR and 95% CI: 1.11 (0.93–1.32)) or “Baseline High then Decrease Pattern” (HR and 95% CI: 1.06 (0.83–1.36)).

The hazards of obesity of adults with different patterns of PEF trajectory among participants with different PEF levels at baseline are presented in Table 3 and Figure 2(B2–B4). Among participants with a PEF lower than the recommendation (<20%) at baseline, compared with the “Stable Pattern”, the “Sudden-Increase Pattern” showed a positive association with obesity (HR and 95% CI: 1.65 (1.13–2.41)). However, no statistically significant association with obesity was observed in the “Moderate-Increase Pattern” (HR and 95% CI: 1.02 (0.82–1.27)) or “Substantial-Increase Pattern” (HR and 95% CI: 1.26 (0.98–1.62)). Among participants who met the PEF recommendation (20–30%) at baseline, compared with the “Stable Pattern”, the “Sudden-Increase then Decrease Pattern” showed a positive association with obesity (HR and 95% CI: 1.59 (1.03–2.46)). No statistically significant association with obesity was found for the “Moderate-Increase Pattern” (HR and 95% CI: 1.02 (0.71–1.47)) or “Substantial-Increase Pattern” (HR and 95% CI: 1.03 (0.71–1.50)). Among participants with a PEF higher than the recommendation (>30%) at baseline, compared with the “Stable Pattern”, negative associations were observed for the “Stable-Decrease Pattern” (HR 95% CI: 0.91 (0.66–1.25)), “Decrease then Increase Pattern” (HR and 95% CI: 0.60 (0.36–1.01)), and “Decrease-but-still-High Pattern” (HR and 95% CI: 0.88 (0.62–1.27)); however, the associations were not statistically significant.

The results of the sensitivity analyses of samples with missing values imputed by using the multiple imputation method did not differ very much from the aforementioned results of the analytic samples, with the direction and magnitude of the association being persistent (online Supplementary Tables S1 and S2).

## 4. Discussion

Our present study estimated the trajectories of the percentage of energy from fat among Chinese adults by using over 20 years of longitudinal data and assessed the association between PEF trajectory with the risk of general obesity. Our results showed no significant association between the PEF level at baseline and the risk of obesity. However, when the PEF increased from normal to higher than the recommendation (>30%), there was a higher risk of obesity, while approaching the upper boundary of the recommendation (30%) but keeping steady did not increase the risk of obesity. In addition, the results of the analyses among participants with different baseline PEF levels showed that, among adults whose PEF levels were lower than the dietary recommendation (<20%) or met the recommendation (20–30%) at baseline, participants with a sudden increase in PEF during the follow-up period had a higher risk of obesity. Our findings indicated that adults should avoid a sudden increase in PEF to prevent obesity.

In the present study, we found that most of the participants’ PEF increased during the follow-up period and participants’ average PEF increased from 24.9% to 33.6% from 1991 to 2011. The increasing trend in PEF observed in our study is consistent with results of other studies in Asian population [32,33]; however, it is different from results of studies in Western population. Studies in the US adults showed that the PEF decreased from 36.6% in 1971 to 33.7% in 2006 and 33.2% in 2016 [15,16]. Among the Australian population, the PEF declined from 35.3% in 1983 to 31.9% in 1995 and 30.9% in 2012 [14]. In the past decades, the Western population’s PEF showed a downtrend, while the Chinese population’s PEF has been increasing and has neared, and even been higher than, the Western population’s level. The upward trend in PEF among the Chinese population might mainly be caused by the transition of food consumption. In China, with the rapid economic and social development since the 1990s, the food supply has become diverse and abundant. Chinese people’s average consumption of edible oil and meat has increased from 29.5 g/d and 58.9 g/d to 42.1 g/d and 89.7 g/d, respectively, from 1992 to 2012, while the consumption of cereals and vegetables has decreased [34]. Chinese people’s dietary pattern has gradually changed from a low-fat, high-carbohydrate characterized traditional Chinese diet to a high-fat characterized Western diet [8].

Our results showed that the trajectory of PEF was associated with the risk of obesity, rather than the PEF level at baseline. Adults whose dietary PEF increased in a short time, regardless of PEF level at baseline, were more likely to become obese. The mechanism might be that the rapid increase in PEF is more likely to lead to fat accumulation in the body. However, no study disclosed the metabolic effect of the rapid dietary fat increase on health. Further studies are needed to evaluate the biological pathways between changes in the PEF and general obesity.

Among participants with a PEF higher than the recommendation (>30%) at baseline, negative associations were observed between PEF decreased and risk of obesity; however, the associations were not statistically significant. It might be because those participants’ PEF levels, while decreased, were still at high levels and not enough to reverse the health risk caused by a previously high PEF. Further follow-up studies are needed to fully reveal the effect of PEF decline on the risk of obesity.

Our study had several strengths. First, our study is based on a large nationally representative prospective longitudinal sample of Chinese adults. Second, the data of weight and height used in our study were measured by investigators rather than participants’ self-reported data. This ensured the reliability and accuracy of the primary outcome variables of the study. Third, participants of our study were followed up for more than ten years with more than four times of repeated dietary measurements, thus making it possible to describe participants’ changes in PEF. With the latent class trajectory analysis, we thoroughly used the longitudinal data to estimate patterns of the change trajectory of the PEF and provided evidence of the association between the long-term fat intake and the risk of obesity. Our study also had some limitations. First, dietary data in the CHNS were collected with three consecutive 24 h dietary recalls. Although the 24 h dietary recall method is a commonly used dietary assessment tool, it is not ideal for assessing long-term dietary habits, and self-reported data might be subject to recall bias. However, the 24 h dietary recall method is suitable to assess the dietary intake of a population with a large sample size [35] and was appropriate for our study’s purpose, i.e., assessing the change trajectory of PEF and its association with obesity. Second, although we controlled for many confounders in the analyses, some unmeasured potential confounders, such as genetic characteristics and intestinal flora, could not be adjusted for. Those confounders may affect the results from this study. Further studies are needed to explore the effects of those unmeasured confounders on the association between long-term fat intake changes and the risk of general obesity. Third, our study assessed only the association between long-term change in the percentage of energy from total dietary fat and obesity; it did not distinguish between the types of fats consumed and their health effects. However, many studies have shown that saturated, monounsaturated, and polyunsaturated fats had different effects on health, especially on the prevention and control of cardiovascular diseases [36,37,38]. In future studies, it is necessary to estimate and compare the associations between the percentage of energy from different types of fats and obesity in order to provide more precise information for disease prevention and control.

## 5. Conclusions

Our study found that Chinese adults’ PEF levels had increased rapidly in the past decades. Adults with a change trajectory that involved a sudden increase to a high level of PEF had a higher risk of general obesity. People who were at low (<20%) or proper PEF levels (20–30%) should avoid increasing their PEF suddenly. Health education and other measures should be taken to prevent a sudden increase in PEF.

## Figures and Tables

**Table 1:** General characteristics of participants by percentage of energy from fat at baseline.

Characteristics at Baseline	Lower than Recommendation (PEF < 20%) 4894 (37.6%)	Met Recommendation (20%≤ PEF ≤ 30%) 4026 (30.9%)	Higher than Recommendation (PEF > 30%) 4105 (31.5%)	Total N = 13,025	*p*
Duration of follow-up, years, mean ± SD	16.41 ± 6.80	13.94 ± 6.97	12.25 ± 6.93	13.96 ± 7.17	<0.001
Cumulative incidence of obesity, *n* (%)	625 (12.8)	483 (12.0)	463 (11.3)	1571 (12.1)	0.095
Socioeconomic characteristics					
Male, *n* (%)	2491 (50.9)	2022 (50.2)	1926 (46.9)	6439 (49.4)	<0.001
Age, years, mean ± SD	39.96 ± 13.92	41.67 ± 14.22	43.44 ± 15.14	41.59 ± 14.48	<0.001
Han nationality, *n* (%)	4153 (84.9)	3585 (89.1)	3653 (89.0)	11391 (87.5)	<0.001
Marital status, *n* (%)					0.220
Married	3883 (79.3)	3221 (80.0)	3219 (78.4)	10323 (79.3)	
Unmarried	755 (14.5)	582 (14.5)	622 (15.2)	1959 (15.0)	
Divorced/separate/widowed	222 (4.5)	193 (4.8)	232 (5.7)	647 (5.0)	
Education level, *n* (%)					<0.001
Illiterate	1630 (33.3)	888 (22.1)	636 (15.5)	3154 (24.2)	
Primary school	1183 (24.2)	822 (20.4)	665 (16.2)	2670 (20.5)	
Middle school	1834 (46.1)	1856 (46.1)	2062 (50.2)	5725 (44.2)	
High school and above	139 (2.8)	391 (9.7)	659 (16.1)	1189 (9.1)	
Income group, *n* (%)					<0.001
Low	3235 (66.1)	1639 (40.7)	1051 (25.6)	5925 (45.5)	
Medium	1291 (26.4)	1653 (41.1)	1770 (43.1)	4714 (36.2)	
High	338 (6.9)	707 (17.6)	1051 (30.3)	2290 (17.6)	
Regions, *n* (%)					<0.001
Northeast	827 (16.9)	836 (20.8)	904 (22.0)	2567 (19.7)	
East Coast	1015 (20.8)	928 (23.1)	1011 (24.6)	2954 (22.7)	
Central	1827 (37.3)	1270 (31.5)	1229 (29.9)	3178 (24.4)	
Western	1225 (25.0)	992 (24.6)	961 (23.4)	4326 (33.2)	
Community type, *n* (%)					<0.001
City	228 (4.7)	693 (17.2)	1234 (30.1)	2155 (16.6)	
Suburb	742 (15.2)	789 (19.6)	870 (21.2)	2401 (18.4)	
Town	506 (10.3)	786 (19.5)	829 (20.2)	2121 (16.3)	
Village	3412 (69.7)	1749 (43.4)	1139 (27.8)	6300 (48.4)	
Lifestyle					
Current smoker, *n* (%)	1603 (32.8)	1333 (33.1)	1195 (29.1)	4131 (31.7)	<0.001
Current drinker, *n* (%)	1751 (35.8)	1465 (36.4)	1477 (36.0)	4693 (36.0)	0.325
Physical activities, *n* (%)					<0.001
Light	851 (17.4)	1588 (39.4)	2199 (53.6)	4638 (35.6)	
Medium	612 (12.5)	728 (18.1)	815 (19.9)	2155 (16.6)	
Heavy	3289 (67.2)	1571 (39.0)	936 (22.8)	5796 (44.5)	
Dietary total energy intake, kcal/d, mean ± SD	2524.26 ± 735.75	2391.68 ± 670.26	2430.54 ± 709.12	2453.56 ± 709.74	<0.001
Percentages of energy from carbohydrate, %, mean ± SD	74.46 ± 6.15	61.84 ± 5.33	49.05 ± 7.43	62.56 ± 12.28	<0.001
Percentages of energy from protein, %, mean ± SD	11.80 ± 2.19	12.22 ± 2.49	12.17 ± 2.84	12.05 ± 2.51	<0.001
Percentages of energy from fat, %, mean ± SD	13.08 ± 4.60	24.99 ± 2.85	38.04 ± 6.70	24.62 ± 11.44	<0.001

PEF, percentage of energy from fat; BMI, body mass index. There were missing data on variables such as marital status, education level, family income, type of community, and physical activity.

**Table 2:** The associations between percentage of energy from fat level at baseline and the risk of obesity: results of Cox proportional hazards regression models with shared frailty.

Baseline PEF Level	Model 1		Model 2		Model 3		Model 4	
	HR (95% CI)	*p*	HR (95% CI)	*p*	HR (95% CI)	*p*	HR (95% CI)	*p*
Met recommended PEF (20–30%)	Reference		Reference		Reference		Reference	
Lower than recommended PEF (<20%)	0.86 (0.77–0.97)	0.014	0.86 (0.76–0.98)	0.020	0.99 (0.87–1.13)	0.923	1.00 (0.88–1.14)	0.999
Higher than recommended PEF (>30%)	1.11 (0.97–1.25)	0.126	1.08 (0.95–1.24)	0.248	0.97 (0.85–1.12)	0.712	0.96 (0.84–1.10)	0.573

PEF, percentage of energy from fat; 95% CI, 95% confidence interval. Model 1: The model has the categorized baseline PEF level as the only risk factor and family as the random effect. Model 2: Further adjusted for sociodemographic factors (gender, age, marital status, nationality, education, family economic level, community type, and region). Model 3: Further adjusted for lifestyle factors, including smoking, drinking, and physical activity. Model 4: Further adjusted for dietary energy intake.

**Table 3:** The associations between change trajectory patterns of percentage of energy from fat and the risk of obesity: results of Cox proportional hazards regression models with shared frailty.

Change Trajectory Patterns of PEF	Model 1		Model 2		Model 3		Model 4	
	HR (95% CI)	*p*	HR (95% CI)	*p*	HR (95% CI)	*p*	HR (95% CI)	*p*
**Overall Participants**								
Baseline Low then Increase Pattern	Reference		Reference		Reference		Reference	
Baseline Normal-Low then Increase-to-High Pattern	1.33 (1.16–1.51)	<0.001	1.18 (1.02–1.36)	0.030	1.17 (1.01–1.36)	0.034	1.18 (1.01–1.37)	0.033
Baseline Normal-High and Stable Pattern	1.42 (1.23–1.65)	<0.001	1.09 (0.86–1.39)	0.455	1.08 (0.85–1.37)	0.521	1.11 (0.93–1.32)	0.250
Baseline High then Decrease Pattern	1.43 (1.15–1.77)	0.001	1.08 (0.91–1.28)	0.396	1.06 (0.89–1.26)	0.494	1.06 (0.83–1.36)	0.640
**Participants with different baseline PEF levels**								
**Baseline PEF <20%**								
Stable Pattern	Reference		Reference		Reference		Reference	
Moderate-Increase Pattern	1.24 (0.64–1.44)	0.623	1.24 (0.66–1.49)	0.679	1.02 (0.82–1.27)	0.902	1.02 (0.82–1.27)	0.893
Substantial-Increase Pattern	1.32 (1.09–1.73)	0.042	1.28 (1.05–1.72)	0.045	1.24 (0.98–1.62)	0.077	1.26 (0.98–1.62)	0.075
Sudden-Increase Pattern	1.83 (1.32–2.53)	0.003	1.81 (1.32–2.54)	0.008	1.65 (1.13–2.42)	0.012	1.65 (1.13–2.41)	0.010
**Baseline PEF at 20–30%**								
Stable Pattern	Reference		Reference		Reference		Reference	
Moderate-Increase Pattern	1. 23 (0.86–1.53)	0.642	1.10 (0.81–1.50)	0.655	1.02 (0.71–1.48)	0.917	1.02 (0.71–1.47)	0.920
Substantial-Increase Pattern	1.11 (0.64–1.83)	0.547	1.09 (0.67–1.77)	0.559	1.04 (0.71–1.51)	0.866	1.03 (0.71–1.50)	0.865
Sudden-Increase then Decrease Pattern	1.74 (1.13–2.68)	0.027	1.70 (1.12–2.59)	0.025	1.59 (1.02–2.47)	0.021	1.59 (1.03–2.46)	0.038
**Baseline PEF >30%**								
Stable Pattern	Reference		Reference		Reference		Reference	
Stable-Decrease Pattern	0.88 (0.56–1.15)	0.664	0.89 (0.60–1.20)	0.608	0.91 (0.65–1.25)	0.555	0.91 (0.66–1.25)	0.551
Decrease then Increase Pattern	0.56 (0.33–0.96)	0.041	0.60 (0.38–0.99)	0.049	0.60 (0.36–1.01)	0.053	0.60 (0.36–1.01)	0.056
Decrease-but-still-High Pattern	0.72 (0.60–1.13)	0.775	0.73 (0.60–1.22)	0.642	0.89 (0.61–1.26)	0.505	0.88 (0.62–1.27)	0.501

PEF, percentage of energy from fat; 95% CI, 95% confidence interval. Model 1: The model has the change trajectory patterns of PEF as the only risk factor and family as the random effect. Model 2: Further adjusted for sociodemographic factors (gender, age, marital status, nationality, education, family economic level, community type, and region). Model 3: Further adjusted for lifestyle factors, including smoking, drinking, and physical activity. Model 4: Further adjusted for dietary energy intake.

## Data Availability

The data were obtained from the CHNS. The original database is available at the website (https://www.cpc.unc.edu/projects/china) (accessed on 21 July 2020).

## Supplementary Materials

The following supporting information can be downloaded at https://www.mdpi.com/article/10.3390/nu14163373/s1. Table S1: The associations between percentage of energy from fat level at baseline and the risk of obesity: results of Cox proportional hazards regression models with shared frailty. Table S2: The associations between change trajectory patterns of percentage of energy from fat and the risk of obesity: results of Cox proportional hazards regression models with shared frailty.
