# Supplementary material for: Association between Long-Term Changes in Dietary Percentage of Energy from Fat and Obesity: Evidence from over 20 Years of Longitudinal Data
Source: Nutrients. 2022 Aug 17;14(16):3373. doi: 10.3390/nu14163373 (PMC9414001; doi:10.3390/nu14163373)
Supplement: Supplementary file 1 [file nutrients-14-03373-s001.zip › nutrients-1859212-supplementary.pdf]

Supplementary Materials

Table S1. The associations between percentage of energy from fat level at baseline and the risk of obesity: results of Cox proportional hazards regression models with shared frailty.*

Baseline PEF Level	Model 1		Model 2		Model 3		Model 4	
	**HR (95% CI)**	***p***	**HR (95% CI)**	***p***	**HR (95% CI)**	***p***	**HR (95% CI)**	***p***
Met recommended PEF (20%–30%)	Reference		Reference		Reference		Reference	
Lower than recommended PEF (<20%)	0.79 (0.69–0.92)	0.012	0.83 (0.72–0.99)	0.038	0.99 (0.86–1.19)	0.954	1.02 (0.92–1.21)	0.998
Higher than recommended PEF (>30%)	1.12 (0.96–1.33)	0.141	1.09 (0.98–1.32)	0.301	0.98 (0.83–1.18)	0.754	0.94 (0.82–1.08)	0.571

* Results of the sensitivity analyses of samples with missing values imputed by using the multiple imputation method. PEF, percentage of energy from fat; 95% CI, 95% confidence interval. Model 1: The model has the categorized baseline PEF level as the only risk factor and family as the random effect. Model 2: Further adjusted for sociodemographic factors (gender, age, marital status, nationality, education, family economic level, community type, and region). Model 3: Further adjusted for lifestyle factors, including smoking, drinking, and physical activity. Model 4: Further adjusted for dietary energy intake.

Table S2. The associations between change trajectory patterns of percentage of energy from fat and the risk of obesity: results of Cox proportional hazards regression models with shared frailty.*

Change trajectory patterns of PEF	Model 1		Model 2		Model 3		Model 4	
	**HR (95% CI)**	***p***	**HR (95% CI)**	***p***	**HR (95% CI)**	***p***	**HR (95% CI)**	***p***
**Overall Participants**								
Baseline Low then Increase Pattern	Reference		Reference		Reference		Reference	
Baseline Normal-Low then Increase-to-High Pattern	1.27 (1.17–1.43)	<0.001	1.22 (1.03–1.42)	0.023	1.17 (1.02–1.36)	0.034	1.17 (1.01–1.36)	0.035
Baseline Normal-High and Stable Pattern	1.38 (1.14–1.68)	0.001	1.22 (0.93–1.56)	0.608	1.12 (0.83–1.44)	0.527	1.07 (0.84–1.35)	0.608
Baseline High then Decrease Pattern	1.36 (1.20–1.56)	<0.001	1.31 (0.92–1.66)	0.540	1.09 (0.89–1.27)	0.499	1.06 (0.89–1.25)	0.540
**Participants with different baseline PEF levels**								
**Baseline PEF <20%**								
Stable Pattern	Reference		Reference		Reference		Reference	
Moderate-Increase Pattern	1.19 (0.61–1.43)	0.623	1.21 (0.65–1.45)	0.679	1.04 (0.85–1.24)	0.902	1.02 (0.87–1.29)	0.893
Substantial-Increase Pattern	1.30 (1.09–1.73)	0.042	1.28 (1.05–1.72)	0.049	1.23 (0.96–1.58)	0.081	1.24 (0.95–1.66)	0.079
Sudden-Increase Pattern	1.81 (1.29–2.55)	0.003	1.81 (1.30–2.52)	0.007	1.62 (1.16–2.39)	0.009	1.62 (1.15–2.40)	0.012
**Baseline PEF at 20%–30%**								
Stable Pattern	Reference		Reference		Reference		Reference	
Moderate-Increase Pattern	1.25 (0.84–1.61)	0.651	1.13 (0.83–1.55)	0.660	1.05 (0.75–1.53)	0.932	1.01 (0.74–1.53)	0.945
Substantial-Increase Pattern	1.09 (0.66–1.81)	0.546	1.08 (0.69–1.80)	0.562	1.03 (0.73–1.57)	0.877	1.02 (0.71–1.52)	0.882
Sudden-Increase then Decrease Pattern	1.79 (1.18–2.71)	0.025	1.72 (1.13–2.62)	0.023	1.63 (1.04–2.46)	0.021	1.60 (1.05–2.46)	0.037
**Baseline PEF >30%**								
Stable Pattern	Reference		Reference		Reference		Reference	
Stable-Decrease Pattern	0.82 (0.52–1.18)	0.661	0.86 (0.55–1.24)	0.612	0.89 (0.61–1.22)	0.552	0.90 (0.68–1.30)	0.549

Decrease then Increase Pattern	0.52 (0.36–0.97)	0.039	0.58 (0.41–0.99)	0.046	0.58 (0.37–1.03)	0.052	0.60 (0.35–1.01)	0.057
Decrease-but-still-High Pattern	0.86 (0.57–1.16)	0.779	0.71 (0.58–1.20)	0.645	0.86 (0.63–1.29)	0.511	0.87 (0.63–1.27)	0.503

* Results of the sensitivity analyses of samples with missing values imputed by using the multiple imputation method. PEF, percentage of energy from fat; 95% CI, 95% confidence interval. Model 1: The model has the change trajectory patterns of PEF as the only risk factor and family as the random effect. Model 2: Further adjusted for sociodemographic factors (gender, age, marital status, nationality, education, family economic level, community type, and region). Model 3: Further adjusted for lifestyle factors, including smoking, drinking, and physical activity. Model 4: Further adjusted for dietary energy intake.
